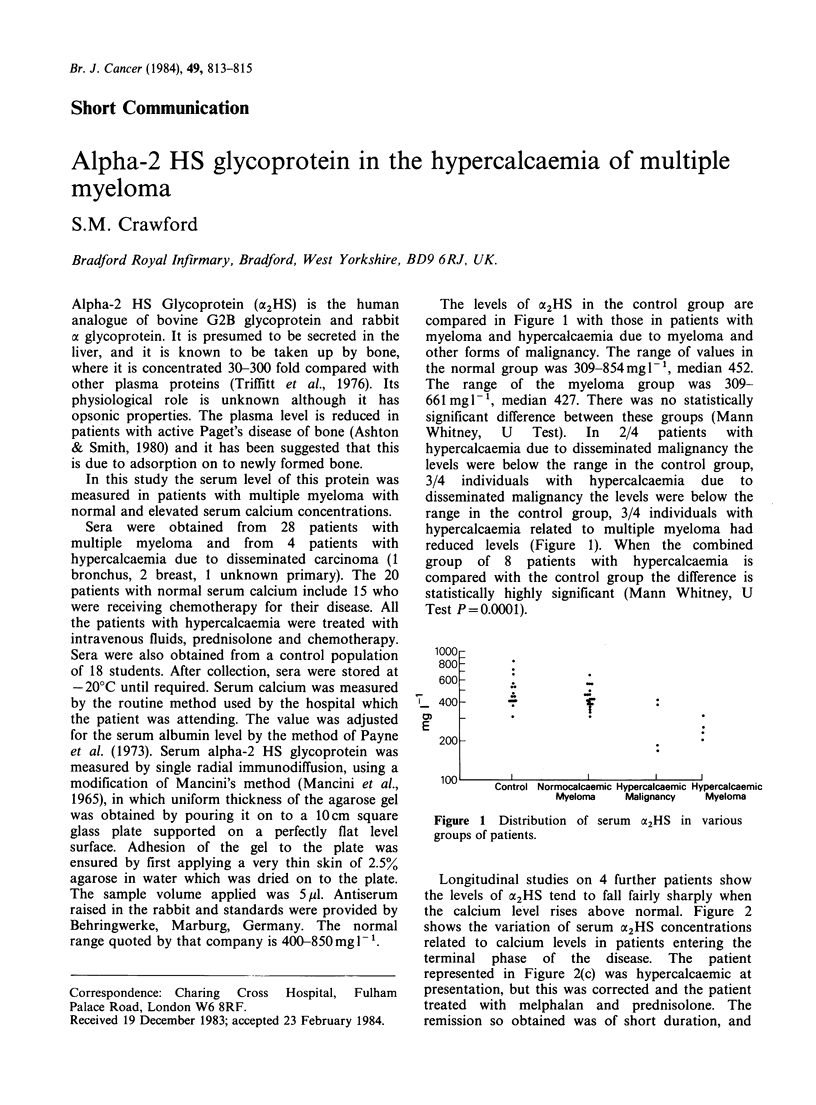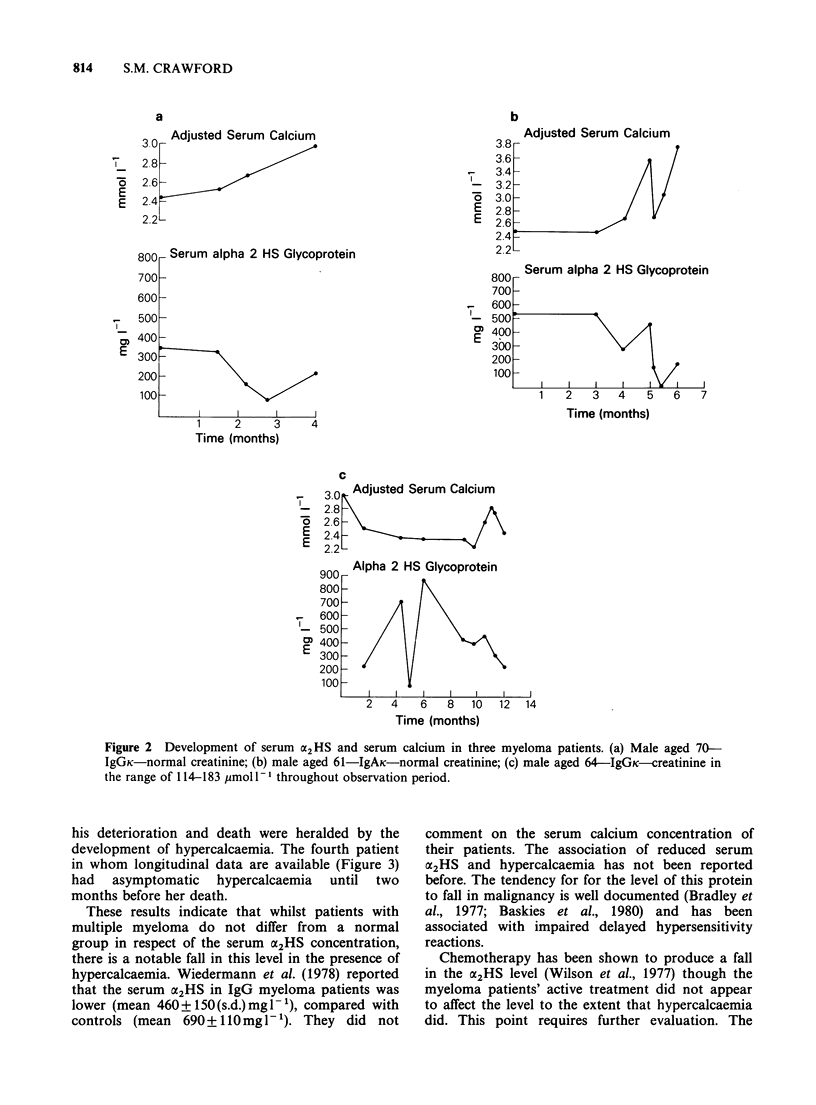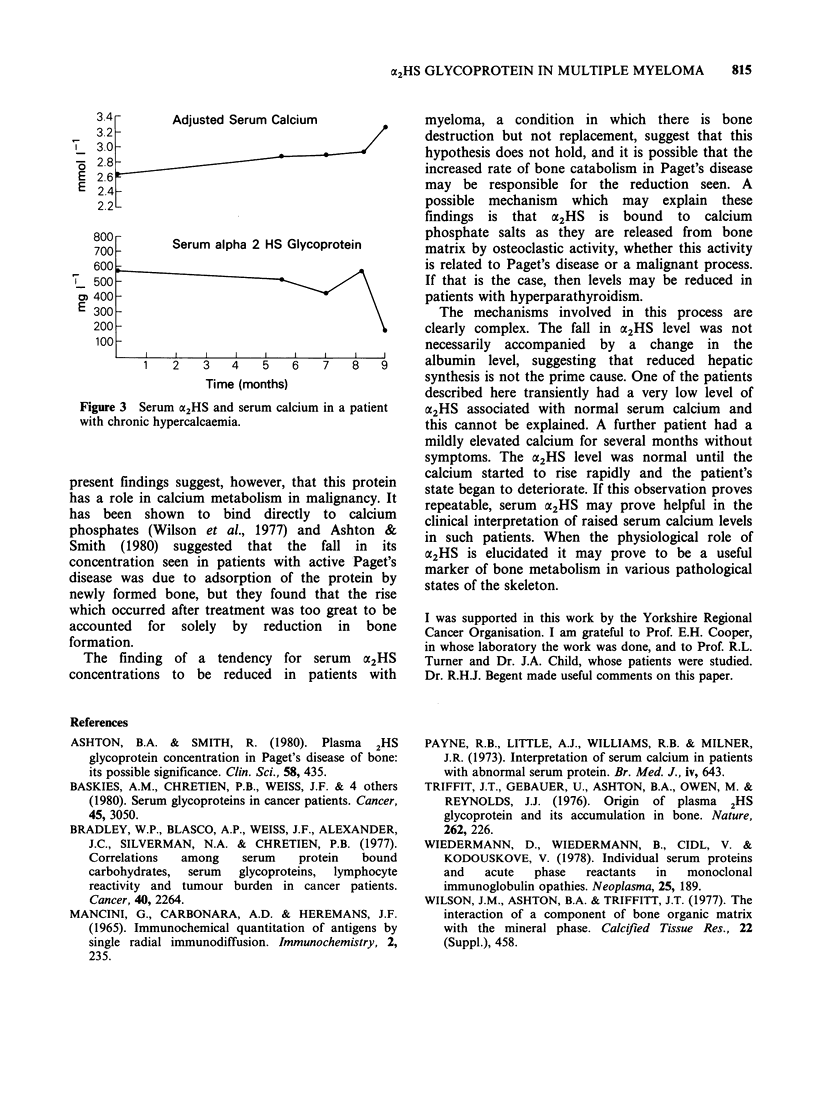# Alpha-2 HS glycoprotein in the hypercalcaemia of multiple myeloma.

**DOI:** 10.1038/bjc.1984.127

**Published:** 1984-06

**Authors:** S. M. Crawford


*Br. J. Cancer* (1984), **49**, 813–815

**Short Communication**

### Alpha-2 HS glycoprotein in the hypercalcaemia of multiple myeloma

S.M. Crawford

*Bradford Royal Infirmary, Bradford, West Yorkshire, BD9 6RJ, UK.*

Alpha-2 HS Glycoprotein ($\alpha_2$HS) is the human analogue of bovine G2B glycoprotein and rabbit $\alpha$ glycoprotein. It is presumed to be secreted in the liver, and it is known to be taken up by bone, where it is concentrated 30–300 fold compared with other plasma proteins (Triffitt *et al.*, 1976). Its physiological role is unknown although it has opsonic properties. The plasma level is reduced in patients with active Paget's disease of bone (Ashton & Smith, 1980) and it has been suggested that this is due to adsorption on to newly formed bone.

In this study the serum level of this protein was measured in patients with multiple myeloma with normal and elevated serum calcium concentrations.

Sera were obtained from 28 patients with multiple myeloma and from 4 patients with hypercalcaemia due to disseminated carcinoma (1 bronchus, 2 breast, 1 unknown primary). The 20 patients with normal serum calcium include 15 who were receiving chemotherapy for their disease. All the patients with hypercalcaemia were treated with intravenous fluids, prednisolone and chemotherapy. Sera were also obtained from a control population of 18 students. After collection, sera were stored at $-20°C$ until required. Serum calcium was measured by the routine method used by the hospital which the patient was attending. The value was adjusted for the serum albumin level by the method of Payne *et al.* (1973). Serum alpha-2 HS glycoprotein was measured by single radial immunodiffusion, using a modification of Mancini's method (Mancini *et al.*, 1965), in which uniform thickness of the agarose gel was obtained by pouring it on to a 10 cm square glass plate supported on a perfectly flat level surface. Adhesion of the gel to the plate was ensured by first applying a very thin skin of 2.5% agarose in water which was dried on to the plate. The sample volume applied was 5 $\mu$l. Antiserum raised in the rabbit and standards were provided by Behringwerke, Marburg, Germany. The normal range quoted by that company is 400–850 mg $l^{-1}$.

Correspondence: Charing Cross Hospital, Fulham Palace Road, London W6 8RF.
Received 19 December 1983; accepted 23 February 1984.

The levels of $\alpha_2$HS in the control group are compared in Figure 1 with those in patients with myeloma and hypercalcaemia due to myeloma and other forms of malignancy. The range of values in the normal group was 309–854 mg $l^{-1}$, median 452. The range of the myeloma group was 309–661 mg $l^{-1}$, median 427. There was no statistically significant difference between these groups (Mann Whitney, U Test). In 2/4 patients with hypercalcaemia due to disseminated malignancy the levels were below the range in the control group, 3/4 individuals with hypercalcaemia due to disseminated malignancy the levels were below the range in the control group, 3/4 individuals with hypercalcaemia related to multiple myeloma had reduced levels (Figure 1). When the combined group of 8 patients with hypercalcaemia is compared with the control group the difference is statistically highly significant (Mann Whitney, U Test $P=0.0001$).

**Figure 1** Distribution of serum $\alpha_2$HS in various groups of patients.

Longitudinal studies on 4 further patients show the levels of $\alpha_2$HS tend to fall fairly sharply when the calcium level rises above normal. Figure 2 shows the variation of serum $\alpha_2$HS concentrations related to calcium levels in patients entering the terminal phase of the disease. The patient represented in Figure 2(c) was hypercalcaemic at presentation, but this was corrected and the patient treated with melphalan and prednisolone. The remission so obtained was of short duration, and

**Figure 2** Development of serum $\alpha_2$HS and serum calcium in three myeloma patients. (a) Male aged 70—IgG$\kappa$—normal creatinine; (b) male aged 61—IgA$\kappa$—normal creatinine; (c) male aged 64—IgG$\kappa$—creatinine in the range of 114–183 $\mu$mol$l^{-1}$ throughout observation period.

his deterioration and death were heralded by the development of hypercalcaemia. The fourth patient in whom longitudinal data are available (Figure 3) had asymptomatic hypercalcaemia until two months before her death.

These results indicate that whilst patients with multiple myeloma do not differ from a normal group in respect of the serum $\alpha_2$HS concentration, there is a notable fall in this level in the presence of hypercalcaemia. Wiedermann *et al.* (1978) reported that the serum $\alpha_2$HS in IgG myeloma patients was lower (mean $460 \pm 150$(s.d.)mg$l^{-1}$), compared with controls (mean $690 \pm 110$mg$l^{-1}$). They did not comment on the serum calcium concentration of their patients. The association of reduced serum $\alpha_2$HS and hypercalcaemia has not been reported before. The tendency for for the level of this protein to fall in malignancy is well documented (Bradley *et al.*, 1977; Baskies *et al.*, 1980) and has been associated with impaired delayed hypersensitivity reactions.

Chemotherapy has been shown to produce a fall in the $\alpha_2$HS level (Wilson *et al.*, 1977) though the myeloma patients' active treatment did not appear to affect the level to the extent that hypercalcaemia did. This point requires further evaluation. The

**Figure 3** Serum $\alpha_2$HS and serum calcium in a patient with chronic hypercalcaemia.

present findings suggest, however, that this protein has a role in calcium metabolism in malignancy. It has been shown to bind directly to calcium phosphates (Wilson *et al.*, 1977) and Ashton & Smith (1980) suggested that the fall in its concentration seen in patients with active Paget's disease was due to adsorption of the protein by newly formed bone, but they found that the rise which occurred after treatment was too great to be accounted for solely by reduction in bone formation.

The finding of a tendency for serum $\alpha_2$HS concentrations to be reduced in patients with myeloma, a condition in which there is bone destruction but not replacement, suggest that this hypothesis does not hold, and it is possible that the increased rate of bone catabolism in Paget's disease may be responsible for the reduction seen. A possible mechanism which may explain these findings is that $\alpha_2$HS is bound to calcium phosphate salts as they are released from bone matrix by osteoclastic activity, whether this activity is related to Paget's disease or a malignant process. If that is the case, then levels may be reduced in patients with hyperparathyroidism.

The mechanisms involved in this process are clearly complex. The fall in $\alpha_2$HS level was not necessarily accompanied by a change in the albumin level, suggesting that reduced hepatic synthesis is not the prime cause. One of the patients described here transiently had a very low level of $\alpha_2$HS associated with normal serum calcium and this cannot be explained. A further patient had a mildly elevated calcium for several months without symptoms. The $\alpha_2$HS level was normal until the calcium started to rise rapidly and the patient's state began to deteriorate. If this observation proves repeatable, serum $\alpha_2$HS may prove helpful in the clinical interpretation of raised serum calcium levels in such patients. When the physiological role of $\alpha_2$HS is elucidated it may prove to be a useful marker of bone metabolism in various pathological states of the skeleton.

I was supported in this work by the Yorkshire Regional Cancer Organisation. I am grateful to Prof. E.H. Cooper, in whose laboratory the work was done, and to Prof. R.L. Turner and Dr. J.A. Child, whose patients were studied. Dr. R.H.J. Begent made useful comments on this paper.

### References

ASHTON, B.A. & SMITH, R. (1980). Plasma $_2$HS glycoprotein concentration in Paget's disease of bone: its possible significance. *Clin. Sci.*, **58,** 435.

BASKIES, A.M., CHRETIEN, P.B., WEISS, J.F. & 4 others (1980). Serum glycoproteins in cancer patients. *Cancer*, **45,** 3050.

BRADLEY, W.P., BLASCO, A.P., WEISS, J.F., ALEXANDER, J.C., SILVERMAN, N.A. & CHRETIEN, P.B. (1977). Correlations among serum protein bound carbohydrates, serum glycoproteins, lymphocyte reactivity and tumour burden in cancer patients. *Cancer*, **40,** 2264.

MANCINI, G., CARBONARA, A.D. & HEREMANS, J.F. (1965). Immunochemical quantitation of antigens by single radial immunodiffusion. *Immunochemistry*, **2,** 235.

PAYNE, R.B., LITTLE, A.J., WILLIAMS, R.B. & MILNER, J.R. (1973). Interpretation of serum calcium in patients with abnormal serum protein. *Br. Med. J.*, **iv,** 643.

TRIFFIT, J.T., GEBAUER, U., ASHTON, B.A., OWEN, M. & REYNOLDS, J.J. (1976). Origin of plasma $_2$HS glycoprotein and its accumulation in bone. *Nature*, **262,** 226.

WIEDERMANN, D., WIEDERMANN, B., CIDL, V. & KODOUSKOVE, V. (1978). Individual serum proteins and acute phase reactants in monoclonal immunoglobulin opathies. *Neoplasma*, **25,** 189.

WILSON, J.M., ASHTON, B.A. & TRIFFITT, J.T. (1977). The interaction of a component of bone organic matrix with the mineral phase. *Calcified Tissue Res.*, **22** (Suppl.), 458.